# A Missed Bilateral, Acute Anterior Exertional Compartment Syndrome of the Leg

**DOI:** 10.7759/cureus.12614

**Published:** 2021-01-11

**Authors:** Fragkiskos A Angelis, Antonios A Koutalos, George Kalifis, Christina Arnaoutoglou, Michael Hantes

**Affiliations:** 1 Department of Orthopaedic Surgery & Musculoskeletal Trauma, University General Hospital of Larissa, Larissa, GRC

**Keywords:** acute, compartment syndrome, exertional, drop foot, decompression

## Abstract

A 26-year-old male athlete presented to our hospital with bilateral leg pain after intense training. He had a history of transient numbness and pain with rigorous exercise but this time pain persisted and drop foot developed. Unfortunately, the diagnosis of acute exertional compartment syndrome was delayed due to late presentation of the patient in our department. He underwent three consecutive surgeries for decompression and debridement. At 13 months follow-up, he is ambulatory with bilateral ankle-foot orthosis. This case presents a bilateral, acute anterior exertional compartment syndrome of the leg and highlights the need for high clinical suspicion and early treatment of the acute exertional compartment syndrome.

## Introduction

Acute compartment syndrome is considered a potentially limb- and life-threatening orthopaedic emergency [1-7]. It can progress rapidly and urgent diagnosis and appropriate treatment are necessary in order to prevent the development of tissue ischemia and necrosis [2,7,8]. Urgent fasciotomies and decompression of the tissue in the affected compartments are the proposed surgical treatment in order to avoid late disability [2,9,10].

Exertional compartment syndrome is a form of compartment syndrome that presents with pain and swelling of the involved compartment [3,4,8]. The symptoms start with exercise and resolve with rest [3,4,8,9]. The treatment is mainly conservative but rarely surgical intervention may be needed [3,8,9]. On the other hand, acute exertional compartment syndrome is rarer [1,3,4,8,9]. It has been associated with sustained athletic or military training and is a surgical emergency [1,3,4,8,9]. Contrary to the exertional compartment syndrome, the symptoms of the acute exertional compartment syndrome do not resolve with rest [4,8] and urgent decompression is warranted.

Here, we describe a rare case of bilateral, acute exertional anterior compartment syndrome of the lower extremity due to vigorous exercise.

The patient was informed that data concerning the case would be submitted for publication, and he provided consent.

## Case presentation

A 26-year-old male amateur soccer player had a three-year history of exercise-induced anterior tibial pain and numbness on both lower legs. During the last three years, the symptoms developed 20-30 minutes after the start of the physical activity and the pain was relieved 15-20 minutes after the cessation of exercise. However, on the day of the patient’s presentation to the emergency room, he had over-trained as he participated in two soccer matches. The first game went in overtime and 38 hours later he participated in another game.

During the second game the patient experienced his typical anterior tibial pain and numbness on both lower legs and by the end of it, the pain became severe. The patient presented to the emergency department of a nearby hospital where rest was recommended and paracetamol together with narcotics were prescribed. During the night, he suffered severe pain unresponsive to pain killers. The next morning (approximately 16 hours after the second game) the patient presented to the emergency department of our hospital and immediate orthopaedic consultation was requested. Inability of ankle dorsiflexion of both legs was noted.

He was not able to walk due to severe, bilateral, lower leg pain and drop foot. Physical examination revealed that the anterior compartment on both lower legs was swollen, tense, and painful on palpation and during the plantar flexion of the ankle and toes. The other compartments were soft and pain-free. Moreover, capillary refill, palpation of dorsalis pedis, and posterior tibialis pulses and sensation were normal. Muscle strength of tibialis anterior muscle was 0/5 in both legs. Some residual activity was noted on the left extensor hallucis longus and extensor digitorum longus. The patient was afebrile but noteworthy laboratory findings included a white blood cell count of 19.900/μL with 90.5% neutrophils, erythrocyte sedimentation rate (ESR) 58mm/hr, and C-reactive protein (CRP) 18,93mg/L. Creatine phosphokinase (CPK) was 14617 IU/L.

**Figure 1:**
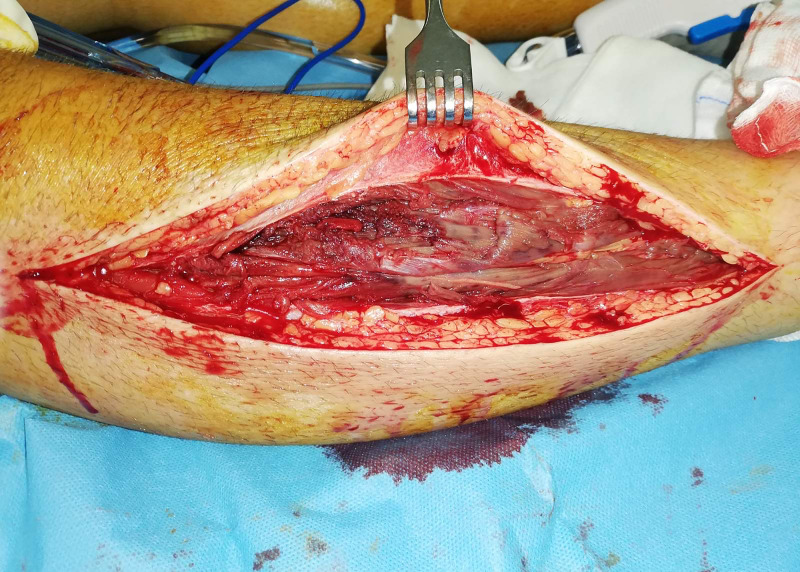
Intra-operative picture of fasciotomy of the anterior compartment of the right leg. Necrosis and micro-vein thrombosis is apparent.

**Figure 2:**
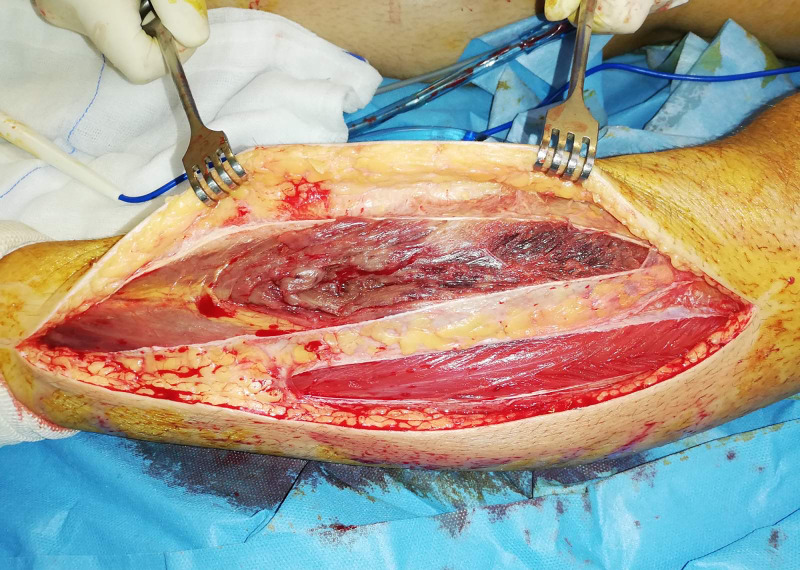
Intra-operative picture of fasciotomy of the anterior and lateral compartment of the left leg. Necrosis and micro-vein thrombosis is apparent in the anterior compartment in contrast to the lateral.

The patient underwent urgent operation for decompression of the compartments of both legs with the two-incision technique. The decision to decompress the compartments late was based on expected pain relief, the fact that some residual muscle mobility and sense was present and, on the need, to prophylactically open all other compartments. The vast majority of both (right and left) anterior compartments were necrotic (Figures 1, 2).

**Figure 3:**
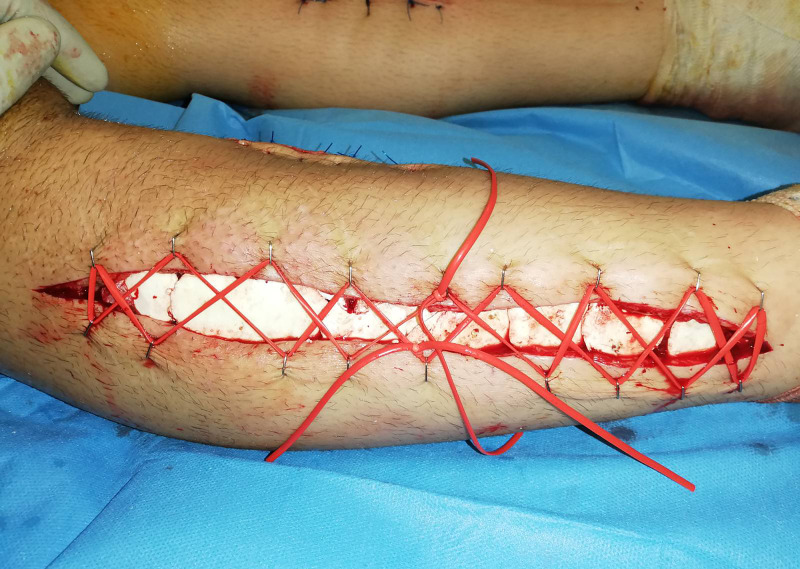
Bead-pouch and shoe-lace technique after the debridement of the right leg.

**Figure 4:**
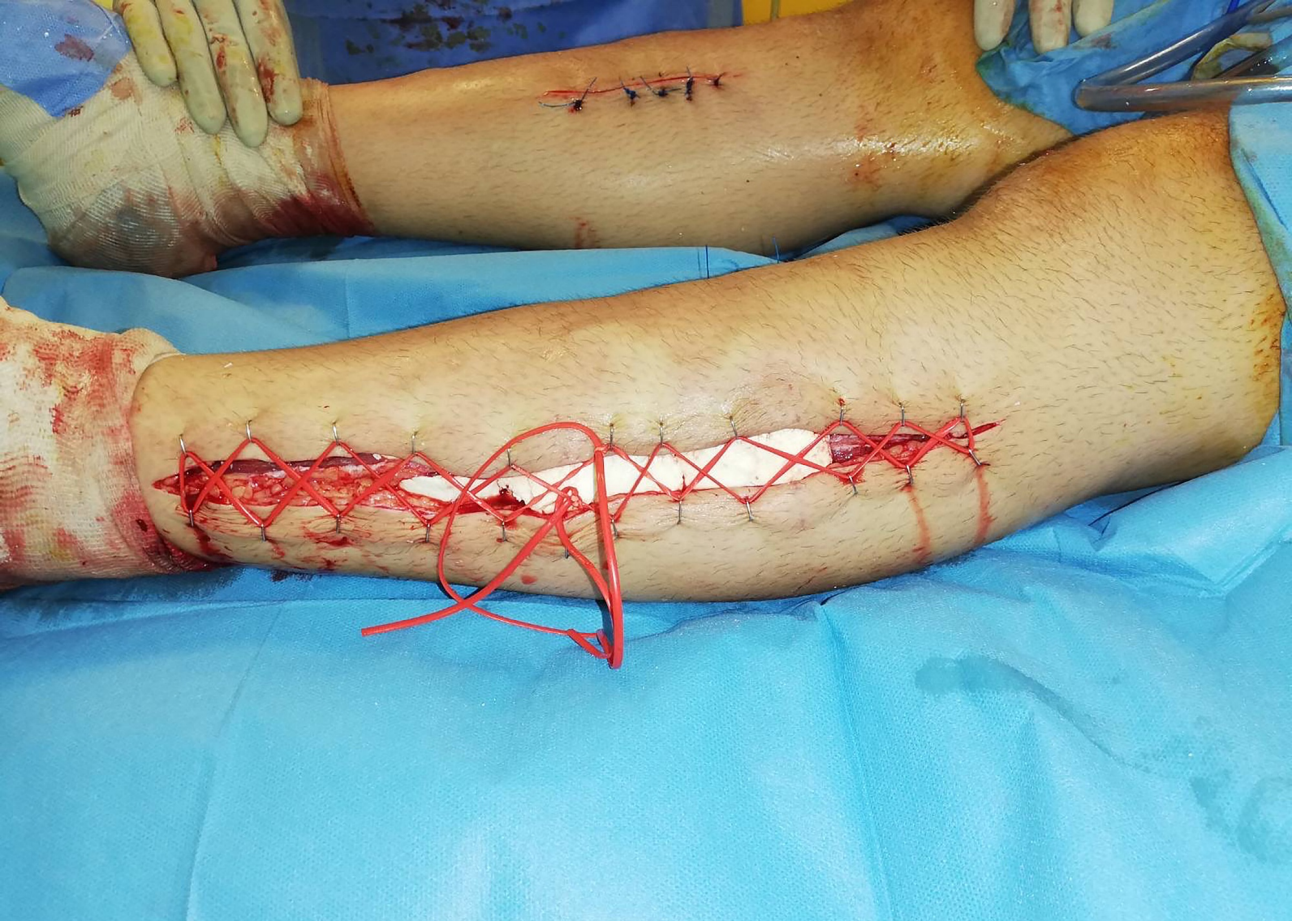
Bead-pouch and shoe-lace technique after the debridement of the left leg.

In addition, there was no response to electrical stimulation and no presence of capillary bleeding. Lateral compartments and the posterior compartments were assessed as normal. Partial debridement, removal of necrotic muscle was performed and an antibiotic bead pouch was placed in the anterior compartments to prevent infection due to late decompression. The shoelace technique was used for skin closure. However, the medial incisions were primary closed. Clips were placed on each side of the wound and mini vessel loops were woven in a crossing way. The wound edges were approximated in a tension-free manner. With each subsequent debridement, the vessel loops were released and tied again. Finally, delayed primary closure of the wound was achieved. However, the medial incisions were primary closed (Figures 3, 4).

Dramatic pain relief was achieved after surgery but no improvement in terms of motion. The patient underwent debridement two additional times. No further clinical improvement was noted.

The patient underwent an event-free hospital stay and eight days later he was discharged. Post-operative rehabilitation protocol included weight-bearing as tolerated, passive mobilization of the ankle, stretching exercises of the Achilles tendon, and electrical muscle stimulation. At the 10-day follow-up the wounds were adequately healed. Thirteen months later the patient was ambulatory with ankle and foot orthosis on both legs and visible extension of the left fourth and fifth toe. However, there was no improvement in the muscle strength of the right leg. He is scheduled for tendon transfers.

## Discussion

We present the case of a patient with bilateral, acute exertional anterior compartment syndrome of the lower extremity due to vigorous exercise. At the last follow-up, the patient has bilateral drop foot and walks with ankle and foot orthoses.

Acute exertional bilateral anterior compartment syndrome of the lower extremity is a very rare condition [3,4]. There are only a few reported cases of bilateral acute exertional compartment syndrome of the lower extremity in the literature. Most cases were due to vigorous exercise, while the two others were attributed to cannabis use and to vascular etiology [2,3,8,9]. One pediatric case was described in a parkour athlete after jogging [11]. Intense training in combination with the presence of sickle cell disease trait has been blamed for acute exertional compartment syndrome [12]. Finally, the syndrome has been described in patients with infective panniculitis [13]. Our patient did not have sickle cell disease. He had a three-year history of exertional compartment syndrome, which progressed to acute syndrome due to sustained training and lack of adequate rest and appropriate treatment.

The exact pathophysiologic mechanism of the exertional compartment syndrome is still largely unknown [1-4,8]. However, it has been described that during exercise the muscle volume can increase up to 20%, and as the muscular compartments have poor compliance, the hydrostatic pressure inside the tissue increases [4,8]. In the acute form, tissue damage and finally muscle necrosis may occur due to the intracellular edema and the restricted venous and lymphatic outflow [4,8]. Myocyte death leads to release of cell contents in the interstitial space, which causes osmotic accumulation of fluid and further increase of intracompartment pressure [14].

Exertional compartment syndrome is a benign condition, however when pain increases despite rest, acute compartment syndrome is suspected and should be treated as such. Endurance training in athletes and being an active military personnel may predispose to chronic exertional compartment syndrome. Sickle cell disease trait may lead a chronic exertional syndrome to decompensate into acute exertional syndrome [12]. The diagnosis mainly based on three signs: pain, paresthesia, and paralysis [15]. Pain is an early sign. It is intense, throbbing and resistant to conservative treatment [3,4,15]. Early paresthesia should be considered an alarm sign and is the result of nerve compression [3,4,15]. Paralysis appears later with sensory and motor deficits, which represent the more progressive and advanced form of paresthesia [3,4,15]. Of note, assessment of compartment pressure is useful in establishing the diagnosis, especially in the obtunded patient [3,4,15]. However, in our patient the diagnosis was quite clear and thus we did not measure the pressure.

Treatment for acute exertional compartment syndrome is surgical fasciotomy. The two-incision technique should be used, with long-enough medial and lateral incisions to completely release all the surrounding compartments [5]. Late fasciotomy is correlated with infection but in this case, we decided to proceed for the above-mentioned reasons [16].

## Conclusions

Here we present a rare case of acute exertional bilateral anterior compartment syndrome of the lower extremity in a young athlete. We hypothesize that the progression to acute exertional compartment syndrome was due to the vigorous exercise and lack of adequate rest and appropriate treatment. Hospital admission, surgical consultation, and observation are recommended for any patient with severe extremity pain after injury or exercise. Repeated clinical assessment every one to two hours would facilitate earlier diagnosis. High clinical suspicion of acute exertional compartment syndrome and early appropriate treatment would prevent the devastating sequelae of this rare clinical entity.
